# Interaction between Bean and *Colletotrichum gloeosporioides*: Understanding Through a Biochemical Approach

**DOI:** 10.3390/plants8090345

**Published:** 2019-09-12

**Authors:** Nilanjan Chakraborty, Kabita Mukherjee, Anik Sarkar, Krishnendu Acharya

**Affiliations:** 1 Department of Botany, Scottish Church College, Kolkata 700006, India; kabitamukherjee95@gmail.com; 2 Molecular and Applied Mycology and Plant Pathology Laboratory, Department of Botany, University of Calcutta, Kolkata 700019, India; anik.tolly@gmail.com (A.S.); krish_paper@yahoo.com (K.A.)

**Keywords:** defense enzymes, disease progression, lignin, lipid peroxidation rate, nitric oxide, proline content

## Abstract

In addition to its role in animals, nowadays nitric oxide (NO) is considered as an emerging signaling molecule in plant systems. It is now believed that NO exerts its pivotal role in various plant physiological processes, such as in seed germination, plant developmental stages, and plant defense mechanisms. In this study, we have taken an initiative to show the biochemical basis of defense response activation in bean leaves during the progression of *Colletotrichum gloeosporioides* (Penz.) Penz. and Sacc. in detached bean leaves. Stages of pathogen penetration and colonization were successfully established in the detached bean leaves. Results showed up-regulation of different defense-related enzymes and other defense molecules, such as phenols, flavonoids, callose, and lignin molecules, along with NO at early stages of pathogen invasion. Although in the later stages of the disease, development of NO and other defense components (excluding lignin) were down-regulated, the production of reactive oxygen species in the form of H_2_O_2_ became elevated. Consequently, other stress markers, such as lipid peroxidation, proline content, and chlorophyll content, were changed accordingly. Correlation between the disease index and other defense molecules, along with NO, indicate that production of NO and reactive oxygen species (ROS) might influence the development of anthracnose in common bean.

## 1. Introduction

Several species of *Colletotrichum* are the sole cause of anthracnose diseases in numerous key annual crops and ornamental plants in temperate and subtropical zones around the world [1]. This includes the anthracnose diseases in cucurbits, onion, bean, cotton, pepper, tomato, and strawberry. Anthracnose can be distinguished by sunken necrotic zones, where dark oblong conidial masses are developed [2]. These symptoms appear in both developing and full-grown plant tissues [3]. The necrotic zones frequently coalesce to form large necrotic areas, generally near the leaf margins [4]. Finally, it leads to withering, wilting, and death of infected plant tissues [5]. Most of the crops are susceptible to more than one species of *Colletotrichum* [6,7]. Various species such as *Colletotrichum falcatum, Colletotrichum gloeosporioides, Colletotrichum truncatum, Colletotrichum sansevieriae, Colletotrichum acutatum, Colletotrichum Capsici* and *Colletotrichum coccodes* have shown severe damage in different plants under changeable environmental conditions. *C. gloeosporioides* (Penz.) Penz. and Sacc., belonging to the family Phyllachoraceae of the division Ascomycota, is one of the most recurrently reported plant pathogens within the genus *Colletotrichum* in India.

Common bean *(Phaseolus vulgaris* L.), one of the main legume grains, is a significant source of protein, vitamins, minerals, and fiber, utilized mainly by poor populations throughout the world [8]. It is also rich in unsaturated fatty acids, such as linoleic and oleic acids [9]. The common bean can also fix atmospheric nitrogen via interaction with *Rhizobium* bacteria. Bean anthracnose is mainly caused by the fungus *Colletotrichum lindenmuthianum* and results in serious crop loss in many parts of the world [10]. However, there are only a few reports available that indicate that *C. gloeosporioides* also causes anthracnose in common bean. Leaves, stems, and pods of bean plants are susceptible to the pathogen. The fungus prefers warm, humid conditions for spreading the anthracnose disease uniformly and effectively [11]. The pathogen primarily invades injured or weakened tissues of plants, where various specialized structures are formed between host and pathogen during infection, that is, conidia, acervuli, setae, and appressoria [12].

Nitric oxide (NO) is a freely diffusible and bioactive gaseous compound, which is a major secondary signal molecule involved in a wide range of pathophysiological processes of plants [13,14,15,16,17,18,19,20]. It is still not clear whether the interaction of both NO and reactive oxygen species (ROS) modulate host cell death during pathogen assault [21]. However, they are involved in modifications of protein phosphorylation patterns, ion fluxes, membrane potential, oxidative cross-linking of plant cell wall proteins, extracellular pH modification, and perturbations in the level of cytosolic Ca^2+^ that triggers the localized over expression of resistance genes [22,23,24,25]. There are several reports indicating reduced NO production inhibits hypersensitive response (HR), programmed cell death (PCD), and accumulation of phytoalexins, events connected with host–pathogen interactions [26,27,28]. Literature also indicates that plant cells accumulate NO in response to infection with bacterial, viral, and fungal pathogens [29,30,31,32,33]. NO, along with ROS, has been linked to plant resistance against various necrotic fungal pathogens, including *Fusarium* [24,25,31]. Early evidence suggested that NO production in tomato plants by application of chitosan strongly reduced the pathogenicity of *Fusarium solani* f. sp. *eumartii* [34]. In connection with *C. gloeosporioides,* pitaya fruit treated with NO showed resistance against anthracnose by up-regulation of phenylalanine ammonia-lyase (PAL), chitinase, peroxidase (PO), polyphenol oxidase (PPO), CoenzymeA ligase, and β-1,3 glucanase enzyme activity, as well as induction of total phenolics, flavonoids, and lignin [35].

In this context, a comparative study on biochemical, histochemical, and enzymatic patterns during *C. gloeosporioides* pathogenesis in detached leaves of common bean has been made and compared with that of healthy uninfected control. Furthermore, those data have been correlated with production of NO to establish its involvement as a key signaling molecule in this host–pathogen combination. As far as our literature survey suggests, this is the first study in which we attempt to show the penetration and establishment of this pathogen on bean leaves, and also find out the status of different defense enzymes, other plant defense molecules, and stress-related compounds during the pathogenesis. This might help in understanding the mechanism of disease development, and also in finding out the strategy for combating this devastating pathogen.

## 2. Results

### 2.1. Pathogenicity Test

**Figure 1:**
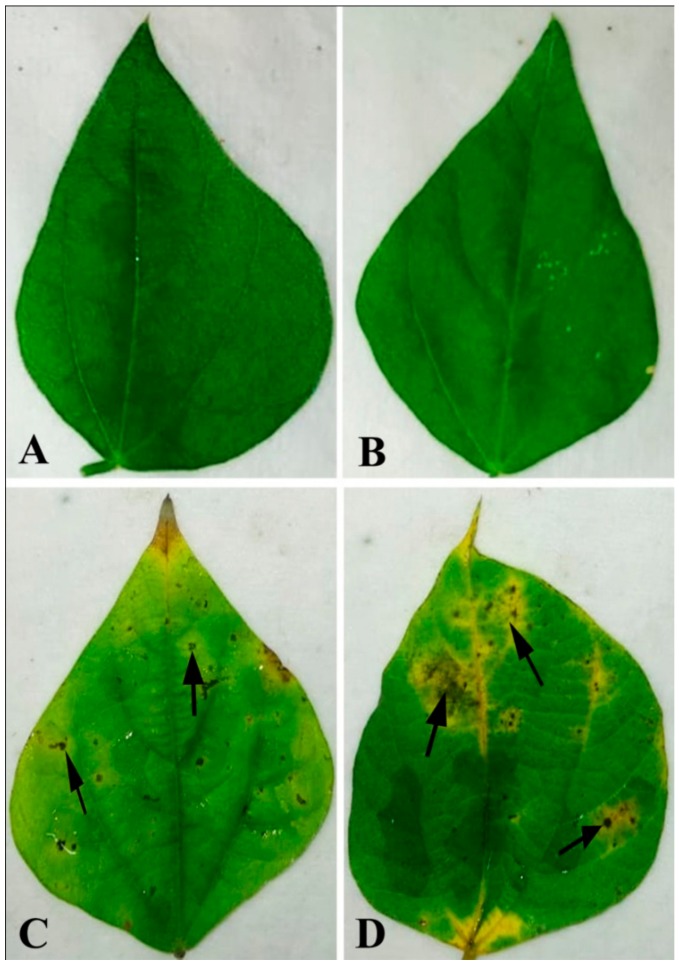
Pathogenicity test on bean leaf. Water-treated control showing no symptoms after (**a**) 2 dai and (**b**) 4 dai. Leaves treated with conidial suspension showing infection zones after (**c**) 2 dai and (**d**) 4 dai. Arrows indicate the site of pathogen infection. Note: dai = days after inoculation.

**Figure 2:**
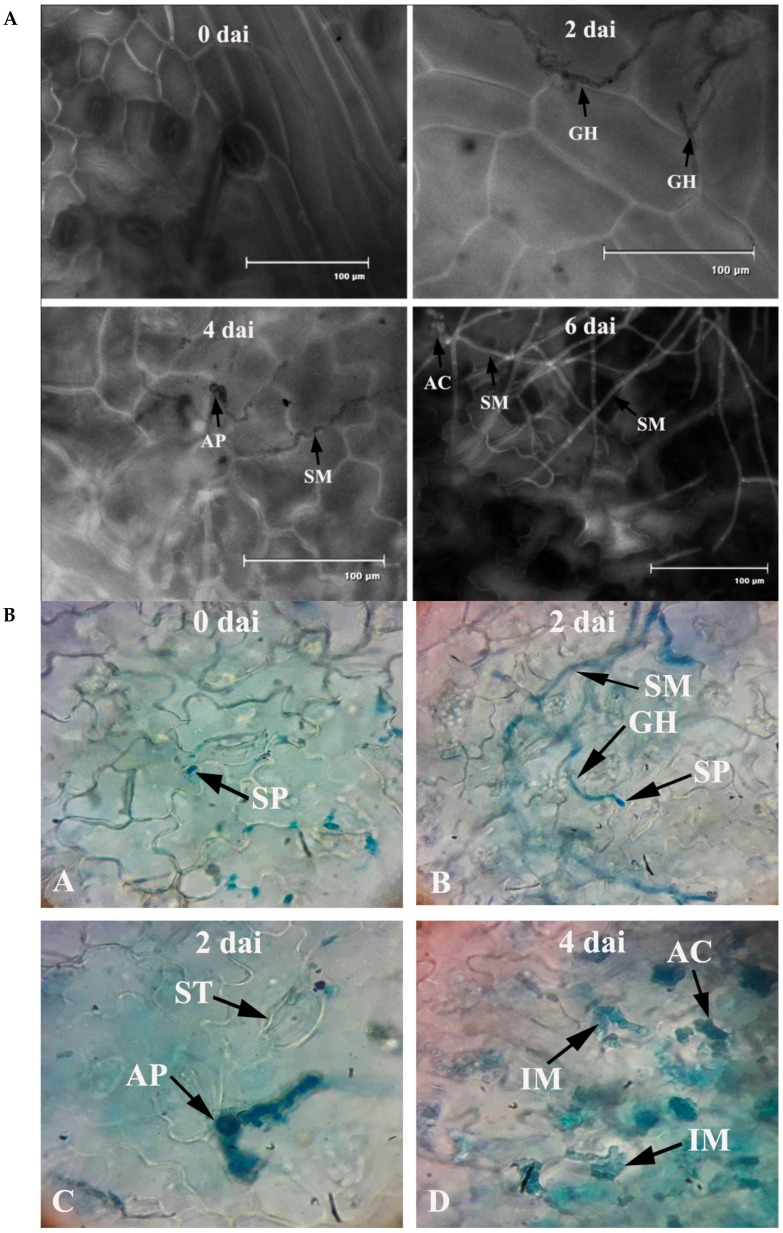
(**A**) Penetration and colonization of leaves of common bean by *C. gloeosporioides*. Note: GH = germinating hyphae; AP = appressorium; SM = surface mycelium; AC = acervuli; dai = days after inoculation. (**B**) Bright field images of penetration and colonization of leaves of common bean by *C. gloeosporioides*. Note: SP = spore; SM = surface mycelium; GH = germinating hyphae; ST = stomata; AP = appressorium; AC = acervuli; IM = intramural hyphae; dai = days after inoculation.

Bean leaves inoculated with fungal conidial suspensions showed typical disease symptoms after two days of incubation (Figure 1). Infection strategy was determined microscopically, which indicates typical *C. gloeosporioides* infection by the formation of the appressoria and acervuli on the leaf surface. The germination of the conidia on the leaf surface was clearly observed (Figure 2). Colorless germinating hyphae were developed from the conidiospore, which ultimately forms an appressorium. The primary infection threads develop from the appressorium and ramify through the sub-cuticular layer and the walls of the epidermis. Then, disease scores were recorded from the infected leaf sample. The pathogen was re-isolated from the leaf and the same morphological characters were recorded. Identical characteristics with the original isolates were observed, hence satisfying Koch’s postulates.

### 2.2. Disease Index (DI)

**Figure 3:**
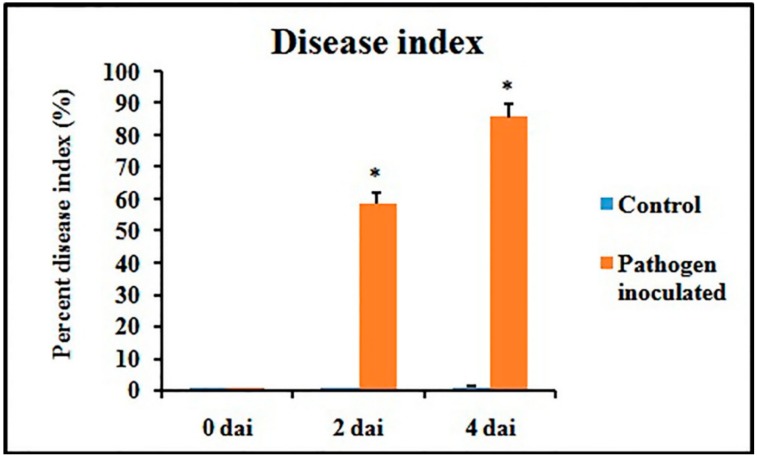
Disease index of pathogen-inoculated detached bean leaf. Values represent mean ± SD of three separate experiments, each in triplicate. * Significant difference between pathogen-inoculated and control sets at *P ˂ 0.05*.

Disease symptoms were observed after 2 dai and 4 dai and disease index (DI)was calculated accordingly. A gradual increase of DI was recorded during the experimental period in pathogen-inoculated detached leaves (Figure 3).

### 2.3. Defense Enzyme Activity

**Figure 4:**
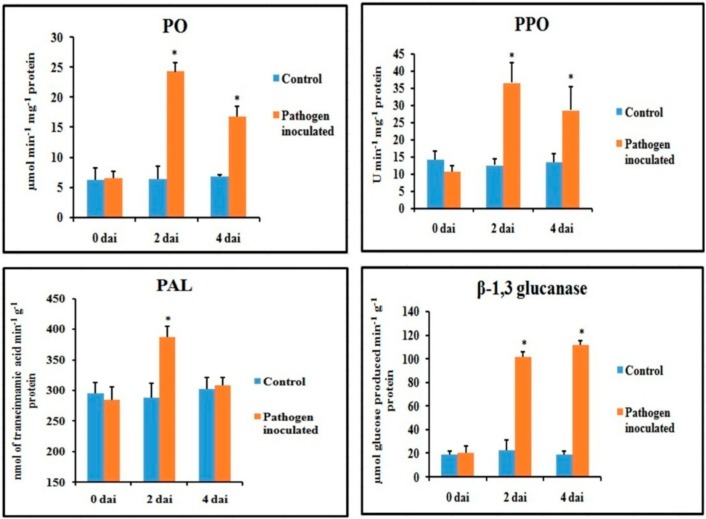
Effect of *C. gloeosporioides* on on the production of defense enzymes in detached bean leaf. Values represent mean ± SD of three separate experiments, each in triplicate. * Significant difference between pathogen-inoculated and control sets at *P ˂ 0.05*.

Defense enzymes were significantly altered in the pathogen-inoculated bean leaves over water-treated control (Figure 4). All of the defense enzymes were significantly higher after two dai and then declined during 4 dai. The β-1,3 glucanase remained higher until 4 dai. A more than 2-fold increase in enzyme activity was observed in peroxidase (PO), polyphenol oxidase (PPO) and β-1,3 glucanase after 2 dai. However, only a 1.3-fold increase in the phenylalanine ammonia-lyase (PAL) activity was observed in the pathogen-inoculated sets over control after 2 dai, which then reduced to basal level at 4 dai. Although PO and PPO activity decreased at 4 dai, they were still significantly higher than that of water-treated control.

### 2.4. Production of Total Phenol and Flavonoid Contents

**Figure 5:**
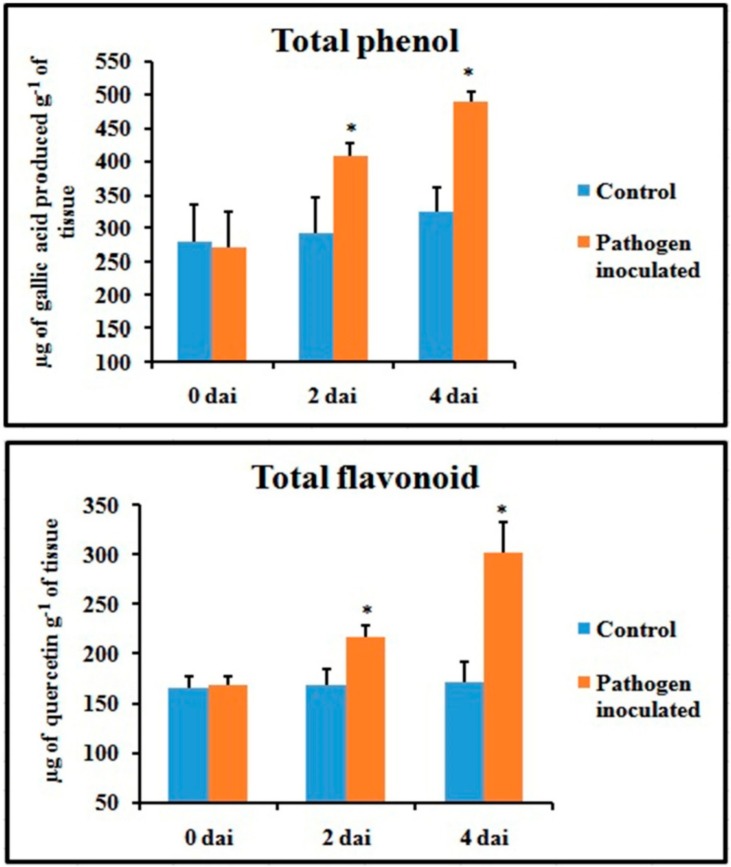
Effect of *C. gloeosporioides* on on the production of total phenols and flavonoids in detached bean leaf. Values represent mean ± SD of three separate experiments, each in triplicate. * Significant difference between pathogen-inoculated and control sets at *P ˂ 0.05*.

Unlike defense enzymes, phenol and flavonoid content of pathogen-inoculated leaves increased gradually (Figure 5) and showed the highest value at 4 dai (1.6-fold and 2.1-fold higher than control, respectively).

### 2.5. Production of NO

**Figure 6:**
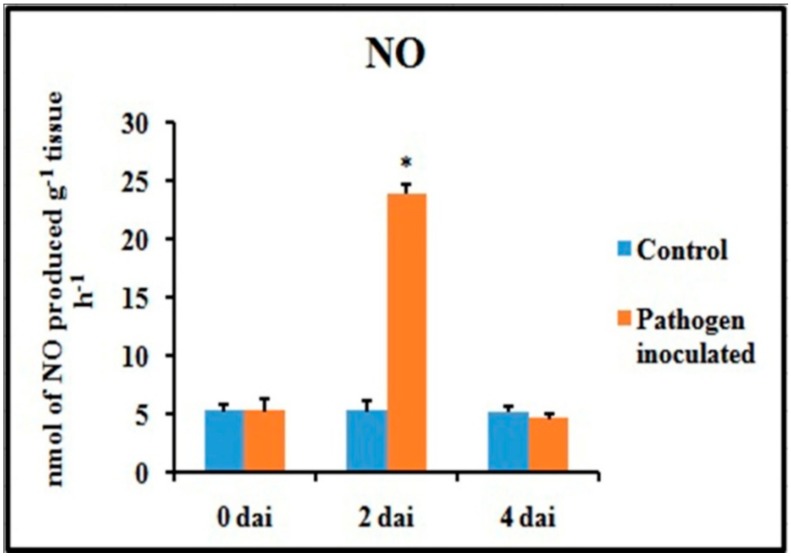
Effect of *C. gloeosporioides* on nitric oxide (NO) production in detached bean leaf. Values represent mean ± SD of three separate experiments, each in triplicate. * Significant difference between pathogen-inoculated and control sets at *P ˂ 0.05*.

An attempt has been made to check the status of NO, a potent bioactive signaling molecule in host-pathogen interaction, at similar time frames (Figure 6). At 2 dai, pathogen-treated leaves showed increased production of NO on leaf tissue and showed more than 4.5-fold increase compared to untreated control leaves. However, at 4 dai, the NO generation was down-regulated to the same basal level as control.

**Figure 7:**
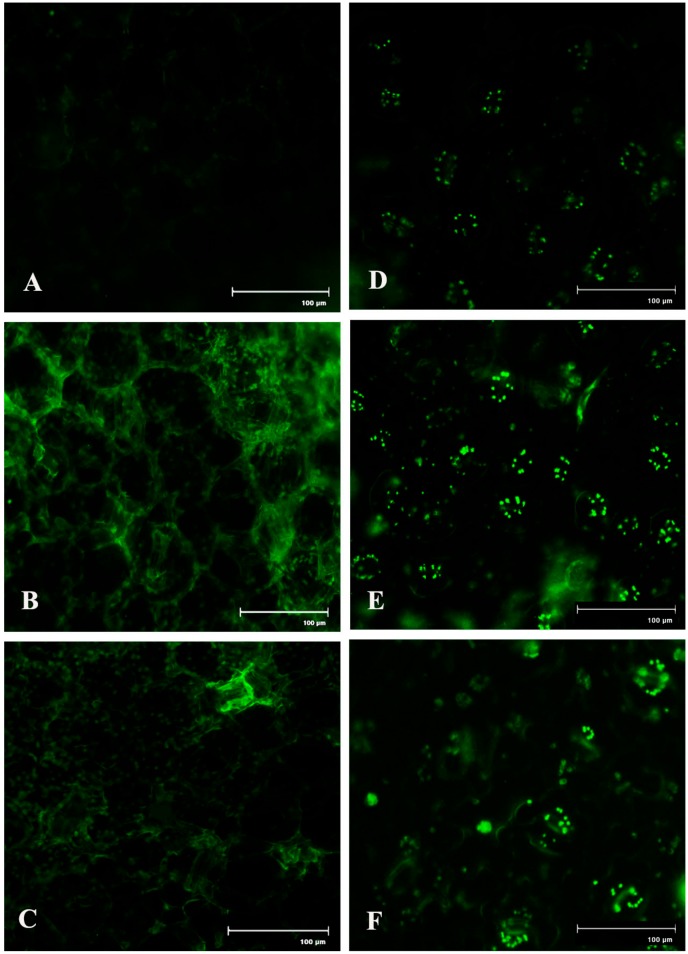
Effects of *C. gloeosporioides* on production of NO in detached bean leaf. Real-time NO generation was detected by green fluorescence in the section of leaf petiole (**A**–**C**) and leaf epidermal cells stained by 4-5 diaminofluorescein diacetate (DAF-2DA): (**A**,**D**) 0 dai; (**B**,**E**) 2 dai; (**C**,**F**) 4 dai.

Spatial formation of NO was further detected by using a NO-specific fluorophore 4-5 diaminofluorescein diacetate (DAF-2DA) on leaf peel and petiole of pathogen-inoculated bean leaves. This forms a green fluorescence when reacting with NO. A similar level of increase in NO production with the higher amount of green fluorescence was observed in both the samples at 2 dai (Figure 7). However, production of NO was reduced to near basal level at 4 dai.

### 2.6. Generation of ROS

**Figure 8:**
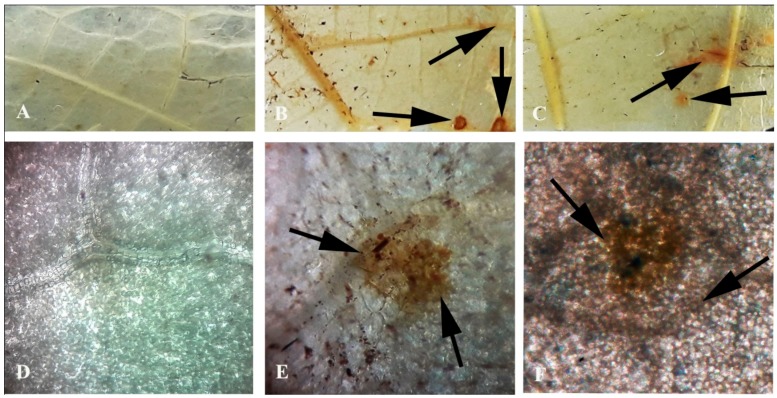
Effect of *C. gloeosporioides* on reactive oxygen species (ROS) generation in pathogen-inoculated bean leaves:(**A**,**D**) 0 dai; (**B**,**E**) 2 dai; (**C**,**F**) 4 dai. Arrows indicate the sites of ROS generation.

Generation of reactive oxygen species in the form of H_2_O_2_ has been monitored in the pathogen-inoculated detached leaves. It was interesting to note that a significant amount of H_2_O_2_ was detected during the gradual progression of the disease symptoms (Figure 8). The production was remarkably high at 4 dai.

### 2.7. Lipid Peroxidation Rate and Proline Content 

**Figure 9:**
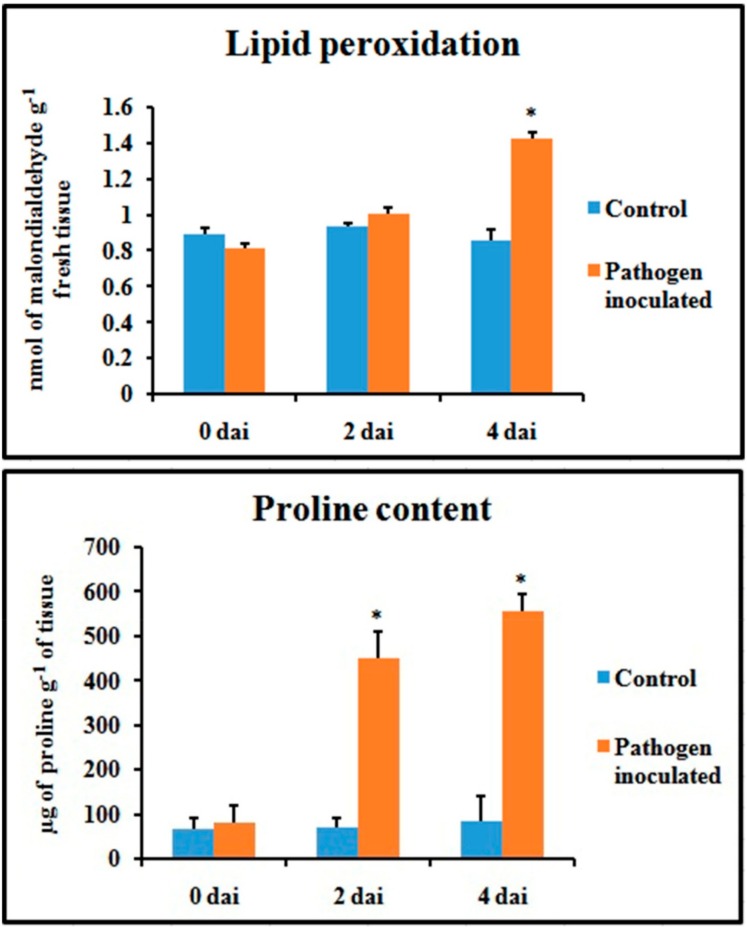
Effect of *C. gloeosporioides* on lipid peroxydation and proline content in pathogen-inoculated bean leaves. Values represent mean ± SD of three separate experiments, each in triplicate. * Significant difference between pathogen-inoculated and control sets at *P ˂ 0.05*.

The extent of oxidative damage by the generation of ROS due to pathogen inoculation was checked as malondialdehyde (MDA) content. Results showed that in bean leaves, the rate of lipid peroxidation was significantly influenced by pathogen inoculation, as it was sharply increased at 4 dai (Figure 9). In the case of proline, it was also gradually increased up to 4 dai, which indicates the stress condition.

### 2.8. Chlorophyll Content

**Figure 10:**
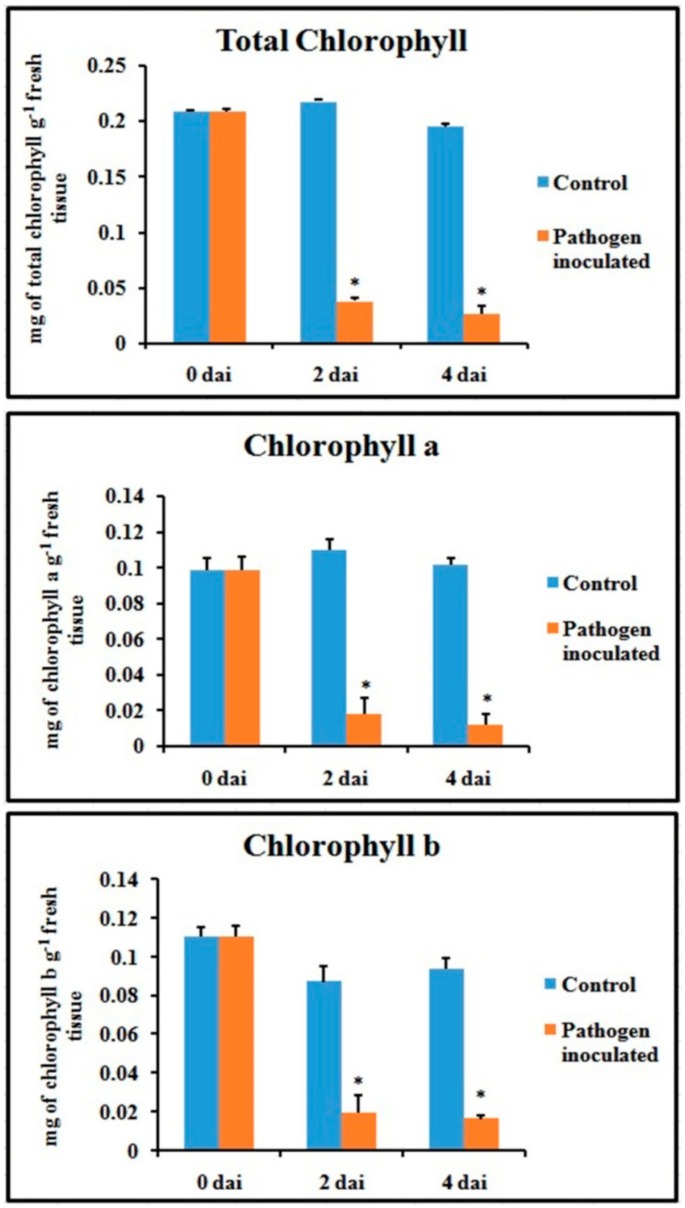
Effect of *C. gloeosporioides* on total chlorophyll content, chlorophyll a content, and chlorophyll b content in pathogen-inoculated bean leaves. Values represent mean ± SD of three separate experiments, each in triplicate. * Significant difference between pathogen-inoculated and control sets at *P ˂ 0.05*.

Total chlorophyll content and fractional chlorophyll a and chlorophyll b content were severely hampered in the pathogen-inoculated leaves (Figure 10). The chlorophyll content was significantly lower during pathogenesis, at least up to 4 dai.

### 2.9. Callose Deposition

**Figure 11:**
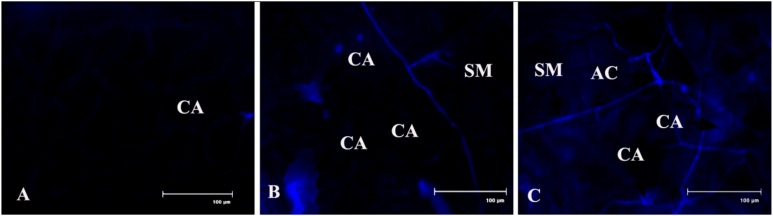
Effects of *C. gloeosporioides* on production of callose in detached bean leaf peel. Real-time callose formation was detected by blue fluorescence in leaf epidermal cells stained by aniline blue: (**A**) 0 dai; (**B**) 2 dai; (**C**) 4 dai. Note: CA = callose; SM = surface mycelium; AC = acervuli.

Callose tissue formation was investigated in the pathogen-inoculated detached bean leaves (Figure 11). It was interesting to note that callose formation was higher after 2 dai than control, but it’s decrease at 4 dai may be because of either an increase of pathogen toxin on the host tissue or an increase of the β-1,3 glucanase enzyme.

### 2.10. Lignin Content

**Figure 12:**
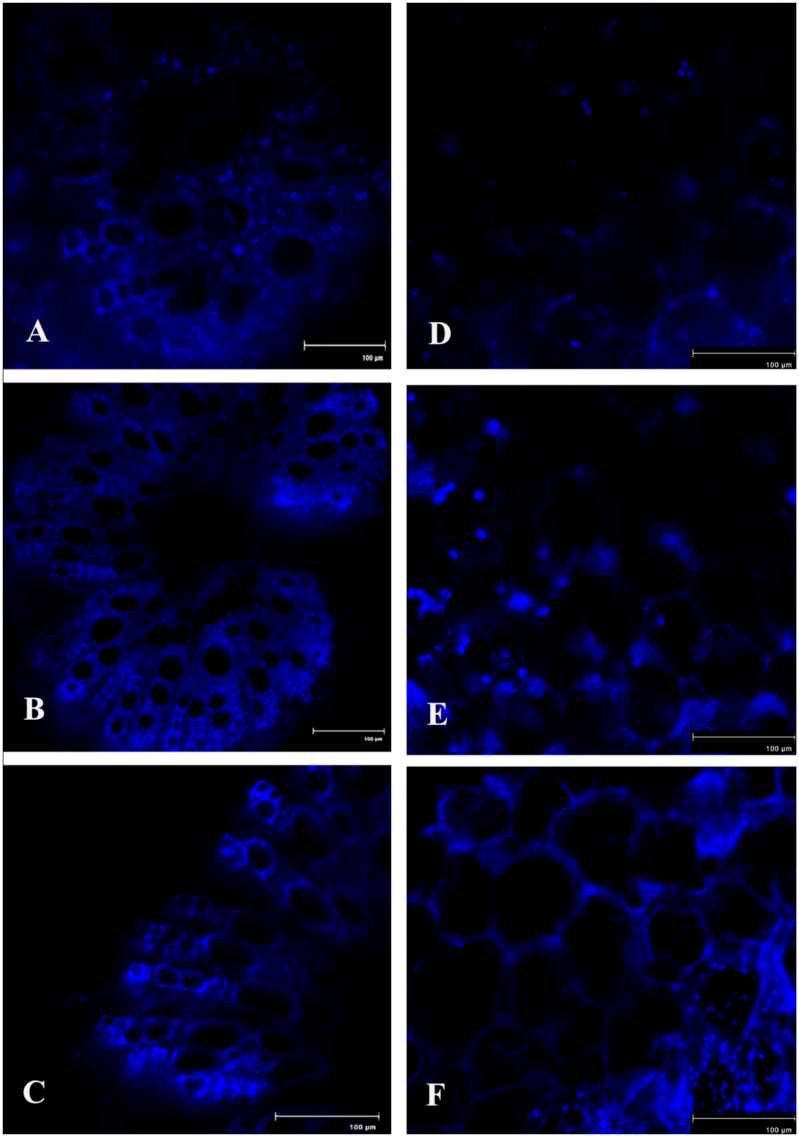
Effects of *C. gloeosporioides* on production of lignin in detached bean leaf petiole (stele and cortex regions). Real-time callose formation was detected by auto-fluorescence in the petiole section: (**A**) 0 dai; (**B**) 2 dai; (**C**) 4 dai.

Production of lignin in the host tissue during pathogen onslaught was also significantly increased up to 4 dai, as investigated in the stele region and cortex region of the infected bean leaf petiole (Figure 12). This indicates the development of systemic acquired resistance (SAR) at the distal part of the pathogen infection.

### 2.11. Effect of NO Donor (SNP) and NO Modulators (C-PTIO and L-NAME) on Defense Response

**Figure 13:**
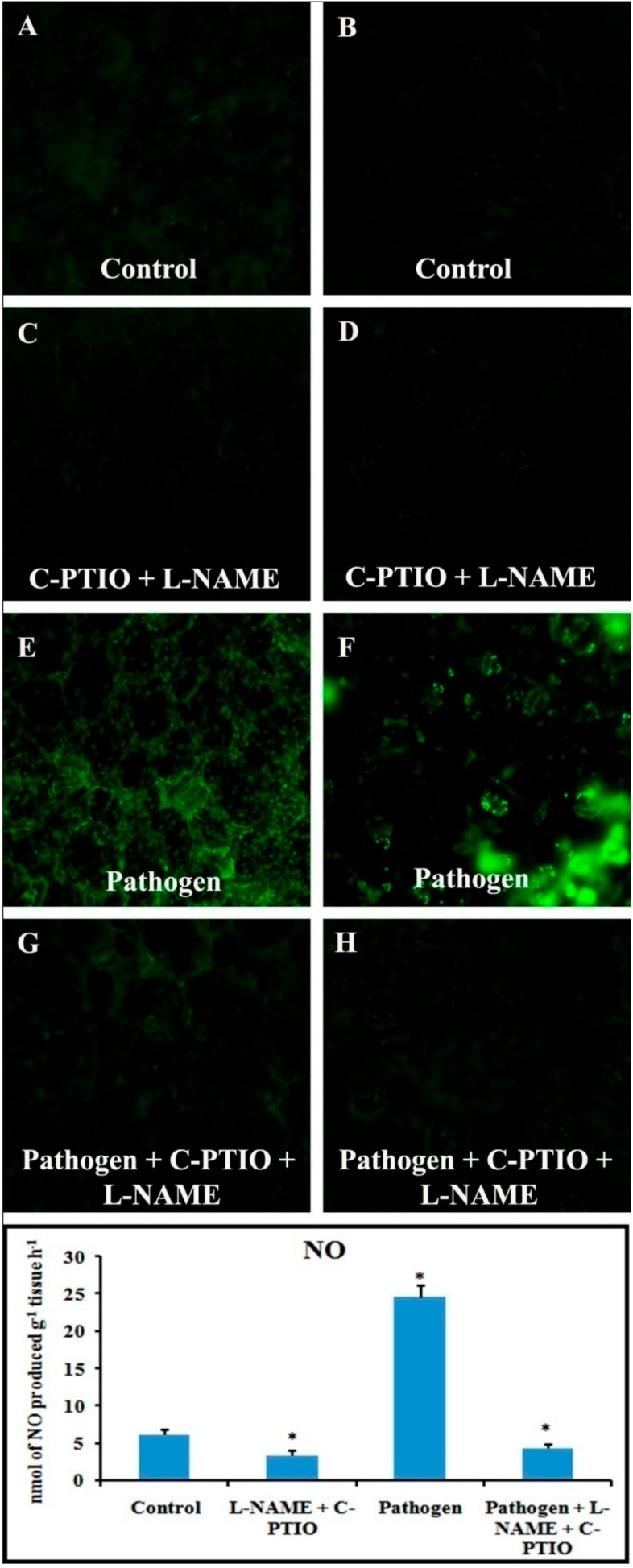
Effects of NO modulators on production of NO in detached bean leaf and petiole. Real-time NO generation was detected by green fluorescence in leaf epidermal cells stained by DAF-2DA: (**A**,**B**) Control; (**C**,**D**) at 2 dai; (**E**,**F**) at 4 dai. Leaf petiole (**A**,**C**,**E**,**G**) and lower epidermis of leaf (**B**,**D**,**F**,**H**). Graph represents spectrophotometric analysis.

**Figure 14:**
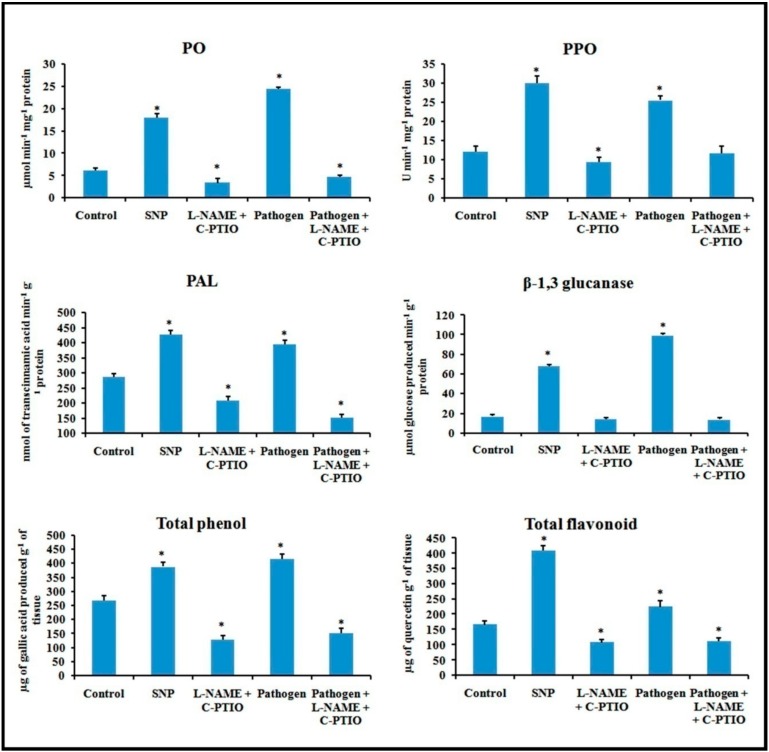
Effect of NO modulators on the production of defense enzymes, phenols, and flavonoids in detached bean leaf. Values represent mean ± SD of three separate experiments, each in triplicate. * Significant difference between pathogen-inoculated and control sets at *P ˂ 0.05*.

Alteration in defense responses, including NO during pathogenesis, have already been stated in the previous results. For further analysis of NO-mediated regulation of plant defense during pathogen progression, leaves were treated with potential NO donor (Sodium nitroprusside, SNP), NO scavenger (2-(4-Carboxyphenyl)-4,4,5,5-tetramethylimidazoline-1-oxyl-3-oxide, C-PTIO), NOS inhibitor (NG-nitro-L-arginine methyl ester, L-NAME), and pathogen combined with NO modulators. Production of NO was altered according to the modulator treatments (Figure 13). NO donor treatment was able to elevate more than two-fold defense responses for all the of defense enzymes i.e., PO, PAL, PPO, β-1,3 glucanase as well as phenols and flavonoids (Figure 14). However, these elevation patterns in defense molecules were compromised and down-regulated during the treatment with the scavenger, NOS inhibitor, and pathogen combined with NO modulators.

## 3. Discussion

In this present study, we examined the defense responses of detached bean leaves against *C. gloeosporioides*. As per our best knowledge, these results provide a novel biochemical basis of *C. gloeosporioides* and bean interaction. Fungal characterization and pathogenecity tests showed the efficacy of the pathogen to infect bean leaves. *Ex vivo* disease scoring pattern showed a time course progression of typical disease symptoms in response to the fungal pathogen. Microscopic observation of diseased detached leaves showed a stepwise manner of pathogen progression inside the host, as observed in a previous study [36].

In recent decades, NO has been considered an important bioactive molecule in various pathophysiological system of plants [37]. The small, lipophillic, gaseous nature makes NO an unavoidable molecule during host–pathogen interaction. It is evidet that NO performs a regulatory role in many host plants to induce innate immunity [18,38,39]. In this study, we showed a time course alteration in NO content in response to pathogen progression. In the detached leaves, NO status was maximum at 2 dai and declined towards 4 dai. The reduced NO content marks the extent of pathogenesis by *C. gloeosporioides*. To confirm the biochemical data of NO status during pathogenesis, real-time NO generation was visualized using 4-5 diaminofluorescein diacetate (DAF-2DA) dye in detached leaves. NO-specific fluorescence in epidermal leaf peels and petiole increased at 2 dai but subsequently decreased at 4 dai. To check whether this augmentation in defense responses is NO-mediated or not, plants were treated with NO donor (sodium nitropruside), NO scavenger (C-PTIO), and nitric oxide synthase (NOS) inhibitor (L-NAME). It was observed that during the period of artificially created NO surplus condition, various defense enzymes and components showed elevation in accumulation and activities. However, under NO-depleted conditions, significant reduction in various defense-related enzymes was observed.

To restrict the progression of a wide range of foreign invading pathogens, plants modulate the activities of different defense-related enzymes, such as PAL, PO, PPO, and β-1,3 glucanase. Among these enzymes, PAL is considered as the starting point of the phenylpropanoid biosynthesis pathway involved in biosynthesis of phenolic compounds [40]. It has been previously established that PAL induction takes part in the biosynthesis of various defense-related secondary metabolites, such as total phenols and flavonoids. In detached leaves, PAL activity is significantly higher at 2 dai. In the case of total phenols and flavonoids, the detached leaves showed an increasing nature until 4 dai. Other defense enzymes such as PO and PPO are associated with elevated defense responses in plants by adjusting the cross-linking of cell walls and accumulation of lignin to hinder the pathogen growth [41]. Our study revealed higher activities of these enzymes in the early stage of pathogen progression and gradual decrease in these enzymes along with disease development in detached bean leaves. Further, we also analyzed the lignin production in detached leaves at the same time points. We found that in both cortical and stele regions, lignin accumulated in higher amounts in response to pathogen infection. This indicates the generation of lignin in the tissues other than the pathogen infection site, marking the development of systemic acquired resistance (SAR) [15]. Higher accumulation of lignin in the later stages of *Colletotrichum* infection was observed in several studies [42].

Another inportant defense-related enzyme, β-1,3 glucanase, hinders fungal growth by destroying the fungal cell wall. This study revealed that the enzyme showed better activity in pathogen-inoculated sets. Callose content was also visualized by aniline blue staining in detached leaves. During the earlier stage of pathogen invasion (at 2 dai), the callose-specific fluorescence was observed to increase, but at the later stage the callose-specific fluorescence declined, which marks the compromise of internal defense responses of plants. Similarly, higher amount of lignin and callose production surrounding the infection zone in the resistant cultivar of *Cucumis melo* L. infected with *Colletotrichum lagenarium* was observed after 48 hours of incubation [42].

It is believed that one of the initial events during host–pathogen interaction is the generation of ROS, which is involved in various defense responses in plants against foreign microbes [43]. In this study, pathogen-inoculated detached leaves showed improved accumulation of ROS (H_2_O_2_) at and around the infection site. ROS accumulation gradually increased and showed maximum intensity at 4 dai; extensive ROS generation may lead to cell death. It has been shown that higher accumulation of ROS accelerates senescence and lowers photosynthetic efficiency in plants [44]. This study showed that the most important photosynthetic pigment, chlorophyll, was reduced with pathogen progression. An important oxidative stress marker, proline, can function as an osmoprotectant and free radical scavenger [45,46,47]. Our results provide evidence that during the pathogen invasion, proline content was significantly higher in pathogen-inoculated leaves until 4 dai. The increasing pattern of proline marks the extent of biotic stress inside the host system. In addition to proline, another useful oxidative stress marker is MDA, which marks the extent of cell membrane damage. Various reports have suggested the increment of MDA during stressful conditions. Our result revealed that along with the pathogen progression in bean leaves, there is a gradual increase in MDA content, which was significantly elevated until 4 dai. However, at this point NO content, along with other defense parameters, were reduced. So, from these initial screenings, it can be stated that NO and ROS function in similar stress responses, but their functioning times are spatially separated.

## 4. Materials and Methods

### 4.1. Plant Material

The experiments were carried out with common bean (*Phaseolus vulgaris* L.). Seeds were collected from the Mycology and Plant Pathology laboratory of the Scottish Church College, Kolkata. Those seeds were planted in earthen pots (6 × 6 × 10 cm) with a potting mixture (clay/vermi-compost/sand, 3:1:1, v/v). Plants were maintained in the experimental garden of the Scottish Church College and watered regularly.

### 4.2. Preparation of Fungal Inoculum 

Pure culture of *Colletotrichum gloeosporioides* (Penz.) Penz. and Sacc. was also obtained from the Mycology and Plant Pathology laboratory of the Scottish Church College, Kolkata. Fungal inoculum was prepared according to a previous study [48]. *C. gloeosporioides* was grown in a Petri dish containing potato dextrose agar (PDA) for 15 days at 25 °C. Subsequently, the Petri dish was flooded with 10 mL of sterile, distilled water and conidia were scraped using a sterile inoculating needle, then filtered through two layers of sterile cloth, and the filtrate containing the conidia was centrifuged at 4000× *g* for 10 min. The supernatant was discarded, and the pellet was re-suspended in 50 mL of sterile, distilled water. The final concentration for conidial suspensions was adjusted to 1 × 10^6^ conidia/mL by counting with a hemocytometer under a light microscope.

### 4.3. Treatment

Mature healthy leaves were collected from the germinated seedlings and washed with sterile, distilled water. Surface water was blotted and placed in a closed chamber on wet blotting paper. One set of leaves was inoculated with spore suspension with the help of a sterile syringe, and the other set of healthy leaves was treated with sterile, distilled water to serve as control. Several spots were inoculated in a single leaf. Each experiment was carried out thrice with six replications for each set.

### 4.4. Pathogenicity Test and Study of Pathogen Invasion

Fresh, healthy 30 days old bean leaves were collected and were surface-sterilized using 0.01% HgCl_2_ solution for 2 min. After two successive washes with sterile, distilled water, the leaves were placed in a sterile Petri dish and kept moist with wet blotting paper. The leaves were then inoculated with 10 μl of conidial suspension (1 × 10^6^ conidia ml^−1^). Simultaneously, a control set was prepared by inoculating the leaf with sterile, distilled water in a similar way. The Petriplates were incubated for 5 days at 25 °C in the dark. The pathogen was re-isolated from diseased leaf and maintained in PDA.

To study the pathogen penetration and colonization process, inoculated leaf pieces approximately 6 × 6 mm were cut and immersed in a clearing solution of absolute ethanol/glacial acetic acid (1:2) overnight to remove chlorophyll [36]. The cleared leaf was mounted on glass microscopic slides in clear glycerin and observed under white and green filters of a Floid Cell Imaging Station microscope (Life Technologies). 

### 4.5. Enzyme Assays

Here, 500 mg of leaf sample was collected from different sets at different time intervals and was extracted with 2 mL of extraction buffer specific for different enzymes. During extraction, 0.1% polyvinylpyrrolidone (PVP) and 20 mL of 1 mM phenylmethylsulfonyl fluoride (PMSF) were added to the buffer: 0.1 M of sodium acetate buffer (pH 5.0) for β-1,3 glucanase; 0.1 M sodium borate buffer (pH 8.7) for PAL; and 0.1 M of sodium phosphate buffer (pH 7.0) for PO and PPO. All of the extraction procedures were conducted at 4 °C. The homogenate was centrifuged at 12,000× *g* for 20 min at 4 °C. The supernatant was used as the crude enzyme source for the enzymatic assay. The supernatant was transferred to a 2 mL eppendorf tube and stored at −20 °C for further use [49,50].

#### 4.5.1. Peroxidase Assay (PO)

PO activity was performed following the method from a previous study [51]. The substrate was prepared by mixing 5 mL of 0.3% H_2_O_2_, 5 mL of 1% guaiacol, and 50 mL of 0.05 M sodium phosphate buffer (pH 6.5). The reaction mixture was prepared with 2.95 mL of substrate and 0.05 mL of enzyme extract, and the absorption change was measured at 470 nm for 3 min. Increased absorbance was recorded and PO activity was expressed as change in the absorption min^−1^ mg^−1^ of protein (E = 26.6 mM^−1^ cm^−1^).

#### 4.5.2. Polyphenol Oxidase Assay (PPO)

PPO activity was assayed according to the method from a previous study [52]. The reaction mixture was prepared with the help of 0.5 mL of crude enzyme extract, 2 mL of 0.1 M sodium phosphate buffer (pH 6.5), and 1 mL of 0.1 M catechol. The mixture was then incubated for 10 min at room temperature. The reaction was stopped by adding 1 mL of 2.5 N H_2_SO_4_. The absorption of formed purpurogallin was measured at 495 nm. The PPO activity was expressed in U min^−1^ mg^−1^ protein (U= change in 0.1 absorbance min^−1^ mg^−1^ protein).

#### 4.5.3. Phenylalanine Ammonia-Lyase Assay (PAL)

PAL activity was determined according to the method from a previous study [53]. Enzyme extract (200 μl) was incubated with 1.3 mL of 0.1 M borate buffer (pH 8.7) and 0.5 mL of 12 mM L- phenylalanine for 30 min at 30 °C. After incubation, the amount of trans-cinnamic acid synthesized was recorded at 290 nm. Enzyme activity was expressed as synthesis of trans-cinnamic acid (in nmol quantities) min^−1^ g^−1^ protein.

#### 4.5.4. β-1,3 Glucanase Assay

The β-1,3 glucanase activity was examined according to the method from a previous study [54]. The reaction mixture was prepared with crude enzyme extract (50 µL) mixed with equal volume of the substrate (1% Laminarin). It was incubated for 1 h at room temperature. Then, the reaction was stopped by adding 300 µL of dintrosalicylic acid reagent followed by boiling for 10 min. The resulting colored solution was diluted with the addition of distilled water to make the total volume 2 mL. The absorption was measured at 520 nm after vortexing. The enzyme activity was expressed as μmol of glucose produced min^−1^ g^−1^ protein.

### 4.6. Estimation of Total Protein Content

The standard Bradford assay [55] was employed to test the protein concentration of each extract. Bovine serum albumin was used as a standard.

### 4.7. Estimation of Total Phenol

Total phenol was measured following the method from a previous study [56]. Here, 250 mg of fresh leaf tissue was ground in 2 mL of 80% methanol and maintained at 65 °C for 15 min. It was then centrifuged at 10,000× *g* for 10 min at room temperature and the supernatant was collected and used to measure the phenol content of the sample. The reaction mixture was prepared by adding 1 mL of crude extract to 5 mL of distilled water and incubated for 2 min. Then, 250 µL of 1 N Folin–Ciocalteu reagent was added to it. The reaction mixture was incubated for 30 min at room temperature. Phenolic content was recorded spectrophotometrically at 725 nm using gallic acid as standard. The amount of total phenol was expressed as μg gallic acid produced g^−1^ tissue.

### 4.8. Estimation of Total Flavonoid Content

Total flavonoid content was estimated by following the method from a previous study [57]. Here, 150 mg of fresh leaf tissue was ground in 2 mL of 80% ethanol and the sample was kept in the dark for 30 min. It was then centrifuged at 10,000× *g* for 5 min at room temperature. The reaction mixture was prepared with 1 mL of crude extract (supernatant) mixed with 4.3 mL of 80% aqueous ethanol, 0.1 mL of 10% aluminum nitrate, and 0.1 mL of 1 M aqueous sodium acetate. The reaction mixture was then kept in the dark for 30 min. After incubation, the absorption was measured at 415 nm. The amount of total flavonoid was expressed as mg g^−1^ of the tissue sample.

### 4.9. Nitric Oxide Estimation (NO)

Production of NO was measured by hemoglobin assay according to the method from a previous study [37]. Leaf tissues of control and treated sets were incubated in a reaction mixture containing 10 mM L-arginine and 10 mM hemoglobin in a total volume of 5 mL of 0.1 M phosphate buffer (pH 7.4). Production of NO was measured spectrophotometrically at 401 nm and NO levels were calculated using an extinction coefficient of 38,600 M^−1^cm^−1^. After 2 h of incubation, NO content in the reaction mixture was measured as nmol of NO produced g^−1^ tissue h^−1^.

Real-time NO production was observed by using membrane permeable flurochrome 4-5 diaminofluorescein diacetate (DAF-2DA) dye [58]. The lower epidermis of the leaf was peeled off and thin sections were prepared from the petiole and placed in a brown bottle containing 1 mL of loading buffer containing 10 mM KCl and 10 mM Tris HCl (pH 7.2) with DAF-2DA at a final concentration of 10 mM for 20 min in dark. Fluorescence was observed with a Floid Cell Imaging Station microscope (Life Technologies) at excitation wavelength of 480 nm and emission wavelength of 500–600 nm. Green fluorescence indicates the production of NO.

### 4.10. In Vivo Detection of Reactive Oxygen Species (ROS) 

The *in vivo* detection of ROS in the form of H_2_O_2_ in treated bean leaves was measured according to the method in a previous study [59]. After treatment with pathogen, the cut ends of the leaves were deeped in a solution containing 1mg mL^−1^ diaminobenzidine (DAB, pH 3.8) and kept for 8 h. After incubation, a central 3 cm^2^ segment of leaves was excised and placed adaxial surface up on filter paper moistened with an ethanol and glacial acetic acid mixture (3:1, v/v) until total removal of chlorophyll. After bleaching, tissues were transferred to water-soaked filter paper for at least 4 h to relax, then finally transferred to paper soaked with lactoglycerol (1:1:1, lactic acid/glycerol/water, v/v) and kept for 24 h. The cleared leaf segments were then observed under light microscope and photographed.

### 4.11. Determination of Lipid Peroxidation Rate

Oxidative damage to leaf lipid was measured by the content of total 2-thiobarbituric acid reactive substances (TBARS) expressed as equivalents of malondialdehyde (MDA) content. TBARS content was estimated using the method from a previous study [60]. Fresh leaf samples (0.2 g) were ground in 5 mL of 0.1% (w/v) trichloroacetic acid (TCA) at 4 °C. Then, they were centrifuged at 12,000× *g* for 5 min. Then, 1 mL of supernatant was added to 4 mL of 0.5% (w/v) thiobarbituric acid (TBA) in 20% (w/v) TCA. Samples were then heated at 90 °C for 30 min. After incubation, the reaction was stopped in an ice bath. Centrifugation was performed at 10,000× *g* for 5 min, and absorbance of the supernatant was recorded at 532 nm and 600 nm. The following formula was applied to calculate malondialdehyde content using its absorption coefficient (ε) and expressed as nmol MDA g^−1^ fresh mass following the formula:

MDA (nmol g^−1^ FM) = [(A532 − A600) × V × 1000/ε] × W

where ε is the specific extinction coefficient (=155 mM cm^−1^), V is the volume of crushing medium, W is the fresh weight of leaf, A600 is the absorbance at 600 nm wavelength, and A532 is the absorbance at 532 nm wavelength.

### 4.12. Estimation of Total Proline Content

Free proline content in the leaves was measured in accordance with the method from a previous study [61]. Here, 200 mg of fresh leaf sample was homogenized in 2 mL of 3% sulphosalicylic acid with the help of a chilled mortar and pestle. The material was centrifuged at 11,000× *g* for 15 min at 4 °C. The reaction mixture was prepared with 1 mL of supernatant, 1 mL of glacial acetic acid, and 1 mL of 0.5% ninhydrin reagent. The reaction mixture was boiled for 30 min, then after cooling, 3 mL of toluene was added to it. The tubes were shaken and the upper layer of toluene was collected using a separating funnel. The absorption of the colored sample was measured at 520 nm against toluene. The amount of proline was calculated by referring to a standard curve of proline and was expressed as µg of proline g^−1^ of tissue.

### 4.13. Estimation of Chlorophyll Content

Total chlorophyll was estimated following Arnon’s method [62]. Here, 500 mg of fresh leaf sample was ground in 4 mL of 80% alkaline acetone (20 mL 0.1 N NaOH) and the extract was centrifuged at 7000× *g* for 10 min at room temperature. The supernatant was collected; the absorbance of the solution was taken at 645 and 663 nm for total chlorophyll, and was calculated with following formula:

Total chlorophyll (mg g^−1^) = 20.2 (D645) + 8.02 (D663) × V/1000 × w

Chlorophyll a (mg g^−1^) = 12.7 (D663) − 2.69 (D645) × V/1000 × w

Chlorophyll b (mg g^−1^) = 22.9 (D645) − 4.68 (D663) × V/1000 × w

where D is optical density, V is final volume of 80% acetone (mL), w is dry weight of sample taken (g).

### 4.14. Measurement of Disease Index (DI)

Disease index was measured every 2 dai for detached leaves according to the method from a previous study [63]. The disease severity was measured on a scale ranging 0–3. The 0–3 scale of the disease severity was classified as follows:

0 = No visible disease symptom on the leaf.
1 = Slight infection, with small spots (≤1 mm).
2 = Moderate infection, with medium spots (1–2 mm).
3 = Extensive infection, with large spots (>2 mm).

Disease index (DI) was calculated using the following formula:

$$\mathrm{Disease}\;\mathrm{Index}\;\left(\mathrm{DI}\right)=\frac{\left(\sum \mathrm{scale}\;\times \;\mathrm{number}\;\mathrm{of}\;\mathrm{spots}\;\mathrm{per}\;\mathrm{leaf}\right)}{\left(\mathrm{Highest}\;\mathrm{scale}\;\times \;\mathrm{total}\;\mathrm{number}\;\mathrm{of}\;\mathrm{spots}\;\mathrm{per}\;\mathrm{leaf}\right)}\times \;100$$

Ten leaves per replication were maintained for each treatment in three sets of replications. Leaves sprayed with sterile water were used as control.

### 4.15. Callose Deposition

Callose deposition in the leaf was measured according to the method from a previous study [64]. Leaves from different treated sets were fixed and distained in acetic acid/ethanol (1:3) until the sample was transparent. After 12 h, the saturated distaining solution was replaced. The leaf sample was then incubated in 150 mM K_2_HPO_4_ for 30 min for washing. After washing, the samples were rinsed with 150 mM K_2_HPO_4_ and 0.01% aniline blue (staining solution), then incubated for 2 h in dark at room temperature. Finally, the sample was mounted in 50% glycerol and observed under fluorescence microscope using DAPI filter. The optimal excitation wavelength was set at 370 nm and the emission wavelength ranges between 490 and 510 nm in the Floid Cell Imaging Station microscope (Life Technologies).

### 4.16. Lignin Production

Lignin production was recorded by the auto-fluorescence microscopy, as described in a previous study [65]. According to the author, lignin has a wide array of fluorescence emission and is excited by UV rays. Lignified cell walls of xylem tissue show auto-fluorescence. Thin hand sections of leaf petiole of different treated leaves were made with a fine blade and mounted in 50% glycerol. Auto-fluorescence of lignin was visualized with the help of a Floid Cell Imaging Station microscope (Life Technologies) under the excitation wavelength 330 nm to 380 nm and emission wavelength between 500 and 530 nm.

### 4.17. Treatment with NO Modulators 

Involvement of NO in the regulation of defense responses during host–pathogen interaction was analyzed by the treatment of NO modulators in the detached leaves of bean. The sets were prepared as follows: Control; Sodium nitroprusside (SNP), 100 mM; 2-(4-Carboxyphenyl)-4,4,5,5-tetramethylimidazoline-1-oxyl-3-oxide (C-PTIO, 100 µM) + NG-nitro-L-arginine methyl ester (L-NAME, 10 µM); Pathogen; Pathogen + C-PTIO (100 µM) + L-NAME (10 µM).Here, SNP acts as a potential NO donor, C-PTIO acts as a NO scavenger, and L-NAME acts as a nitric oxide synthase inhibitor. 

Inhibitor and scavenger were used 8 h prior to the pathogen inoculation. Treatment of same-aged plants with distilled water served as control. Each experiment was carried out with three replications and ten plants at a time. After 48 h, the treated leaves were tested for the abovementioned defense enzymes, phenols, and NO generation.

### 4.18. Statistics

All data presented were analyzed by Student’s t-test using Statistical Package for the Social Sciences (SPSS) software version 20, and in all cases, results are the mean ± standard deviation (SD) of at least six individual experimental data, each in triplicate. Values of *P ˂ 0.05* were considered statistically significant.

## 5. Conclusion

This study establishes that the fungus has detrimental phytotoxic effects in the physiology of common bean. From the above study it can be concluded that depleted NO might be the trademark of successful pathogenesis of the fungus *C. gloeosporioides* in bean plants. Our results showed the positive correlation of NO in the up- and down-regulation of defense-related enzymes and other defense molecules. On the other hand, production of ROS at different time points might also influence disease development. Further research work is needed to reveal the interplay between ROS and NO at the molecular level. This study provides a glimpse into the initial biochemical screening of bean and *C. gloeosporioides* interaction, which could open up a lot of new opportunities in understanding the host–pathogen relationship.
